# Real-World Navigation With Application: Characteristics of Gaze Behavior and Associated Factors in Older Adults

**DOI:** 10.1093/geroni/igad108

**Published:** 2023-09-21

**Authors:** Suguru Shimokihara, Michio Maruta, Gwanghee Han, Yuriko Ikeda, Taishiro Kamasaki, Yuma Hidaka, Yoshihiko Akasaki, Takayuki Tabira

**Affiliations:** Graduate School of Health Sciences, Kagoshima University, Kagoshima, Japan; Research Fellowship for Young Scientists, Japan Society for the Promotion of Science, Tokyo, Japan; Visiting Researcher, Faculty of Medicine, Kagoshima University, Sakuragaoka, Kagoshima, Japan; Department of Health Sciences, Nagasaki University Graduate School of Biomedical Sciences, Nagasaki, Japan; Visiting Researcher, Faculty of Medicine, Kagoshima University, Sakuragaoka, Kagoshima, Japan; Department of Occupational Therapy, School of Health Sciences at Fukuoka, International University of Health and Welfare, Fukuoka, Japan; Department of Occupational Therapy, School of Health Sciences, Faculty of Medicine, Kagoshima University, Kagoshima, Japan; Graduate School of Health Sciences, Kagoshima University, Kagoshima, Japan; Department of Rehabilitation, Medical Corporation, Sanshukai, Okatsu Hospital, Kagoshima, Japan; Department of Rehabilitation, Tarumizu Central Hospital, Tarumizu, Japan; Department of Occupational Therapy, School of Health Sciences, Faculty of Medicine, Kagoshima University, Kagoshima, Japan

**Keywords:** Application, Gaze behavior, Mobile device, Navigation, Real world

## Abstract

**Background and Objectives:**

Advancing age might impair real-world navigation ability. The use of mobile devices by older adults has grown rapidly in recent years. Navigation applications (apps) in mobile devices may facilitate the freedom of outings for older adults. Our aim is to investigate age-related differences in real-world app-based navigation walking in terms of accuracy, efficiency, and gaze behavior; and to explore clinical factors associated with navigation walking in older adults.

**Research Design and Methods:**

A total of 20 community-dwelling older adults and 16 young adults completed a route navigation task using a navigation app while recording their gaze behavior. Outcomes were compared in both groups and a general linear regression was used to explore clinical factors associated with app-based navigation walk in older adults.

**Results:**

Compared with young participants, older participants had more stops and root errors and less fixation time, smaller amplitude of saccades. Additionally, older adults were more likely to glance at their smartphones while app-based navigation walking. Furthermore, gait speed and the following assessment scores were significantly associated with navigation walking in older adults: Mini-Mental State Examination, Life-Space Assessment, and the short version of the Mobile Device Proficiency Questionnaire.

**Discussion and Implications:**

For app-based navigation walks, differences in accuracy and gaze behavior were found to exist with age. Additionally, efficient real-world navigation walks in older adults require the extent of life space and proficiency with mobile devices, along with walking speed and cognitive function. It is possible that age-related functional decline, such as the visual field and shifting attention between mobile devices and the real world, may have influenced the results. The study also suggests the need to understand the level of proficiency with mobile devices so that older adults can continue to go out freely. These findings give the basis for providing older adults with appropriate navigation assistance.

**Translational Significance:** Real-world navigation ability is known to decline with age. Many older adults are now using mobile devices, but little is known about their accuracy and gaze behavior while using navigation applications. Our study revealed that older participants exhibited more stops and errors, smaller saccade angles and fixation durations, and glanced at smartphones more frequently than younger participants during navigation tasks. Efficient navigation was associated not only with cognitive and physical function but also with life space and mobile device proficiency. Our findings provide valuable insights for enhancing the use of technology by older adults and improving their navigation in the real world.

## Background and Objectives

Navigation involves the ability to orient and locate oneself in the real-world, allowing for movement from one place to another (Wolbers & Hegarty, 2010). Navigation in the real-world is a fundamental cognitive function requiring multiple strategies and processing levels (Boccia et al., 2014; Epstein, 2008). Navigation has been categorized as an egocentric or allocentric strategy (Ekstrom et al., 2014). An egocentric navigation strategy is a type of navigation strategy that is based on direction (left–right) responses and actions independent of environmental cues (Wolbers et al., 2004). This strategy is generally used when the same route is followed many times (Hartley et al., 2003). An allocentric navigation strategy is based on using environmental cues, such as landmarks, to navigate through space. This strategy is used when moving along a lesser-known or novel route (Coughlan et al., 2018). These two strategies play a pivotal role in a person’s ability to successfully maintain mobility in the environment (Spiers & Barry, 2015).

Navigation strategies suitable for an individual depend on their sex and age (Boone et al., 2018; Lester et al., 2017), and navigation performance declines with age (Kirasic, 1991; Moffat, 2009). Older adults have reported navigation difficulties and avoiding unfamiliar routes and locations (Burns, 1999). This situation has led to the development of the use of the technology as a navigation aid for older adults as one of the research areas. Werner et al. (2018) showed that navigation assistance using robotic rollators improves navigation in real-life settings. Note that due to the size and weight of the devices, it is not expected to be easy to use their devices on a daily basis. On the other hand, our preliminary study reported that assistance using navigation apps on mobile devices achieved more efficient real-world navigation than maps and pictures with text instructions (Shimokihara et al., 2021). The use of mobile devices such as smartphones and tablets has increased rapidly even among older adults (Faverio, 2022; Ministry of Internal Affairs and Communications, 2022). This means that app-based navigation aids may be the most accessible navigation aids for older adults today. The feasibility of a navigation app as a navigation aid for older adults was demonstrated, but the study did not account for age-related differences in navigation performance using apps.

A new approach to assessing real-world navigation ability has been investigated. Irving et al. (2018) analyzed gaze behavior to assess real-world navigation ability and showed that the number of total and visual exploration saccades significantly increased during spatial navigation tasks. This result emphasizes that recording gaze behavior is a feasible method to measure navigation ability in the real-world, which is close to everyday situations. Measurement of gaze behavior is now used in many studies on a wide variety of subjects (Feld & Plummer, 2019; Joshi et al., 2021; Walsh & Snowball, 2023). The measurement environment ranges from the laboratory to the living environment (D’Innocenzo et al., 2022; Ladouce et al., 2022; Lee et al., 2022; Y. Li et al., 2022; Park et al., 2022). Modern eye-tracking techniques can easily detect the participant’s gaze during navigation and are useful as reproducible parameters. However, previous work has focused only on gaze behavior during navigation. The effects of navigation aids on gaze behavior have not been studied in depth. From a safety and feasibility perspective, especially for older adults, it is important to investigate how the use of navigation aids is associated with the parameters of an individual’s gaze behavior.

Therefore, we hypothesized that by comparing the navigation walking ability and gaze behavior of older adults with young adults using an app-based navigation aid, we could identify characteristics of gaze behavior and navigation aid useability that are specific to older adults. In addition, an exploration of the factors associated with app-based navigation walking ability in older adults is expected to provide useful insights to assist older adults in using their mobile devices to go out freely.

The objectives of this study were twofold:

1. To compare the real-world app-based navigation walk in terms of efficiency, accuracy, and gaze behavior between the older and young adults, with regard to characterizing the app-based navigation performance of older adults.
2. To explore the clinical factors related to the efficiency of real-world navigation walking with apps in older adults.

This study is expected to provide the basic knowledge necessary for older adults to adapt navigation apps in mobile devices—the use of which is expected to increase—to their daily lives.

## Method

### Study Population

Sample size calculation was conducted before the study using G* Power Software (ver. 3.1.9.4). Considering the *t*-test family, α = 0.05, effect size = 0.90, and power = 0.80 for normally distributed data. The effect size was set with reference to the values reported in previous studies of age-related gaze behavior (Chaby et al., 2017; Domínguez-Zamora et al., 2020; Keller Chandra et al., 2011). In these conditions, the sample size required to complete the study was 32 people total, and 16 people for each group.

The community-dwelling older adults were recruited from a temporary employment agency for seniors (Silver Human Resources Centre [SHRC]). They were aged 65 years or older and selected by staff at the SHRC, based on the inclusion criteria, and recruited using an email or bulletin board. The inclusion criteria were as follows: (a) no visual or hearing disability, (b) no previous neurological diseases, and (c) did not need help while walking. The healthy young participants were recruited via the on-campus bulletin board at Kagoshima University. Both young and older adults were excluded if they needed an assisted-walking device such as a cane or walker, or if they had a history of cataracts, glaucoma, or other ophthalmologic diseases. All participants lived independently without assistance for daily activities. All participants received 3,000 Japanese yen (approximately USD $21) for their participation. This study was approved by the Kagoshima University (Faculty of Medicine) Ethics Committee (Ref. No.210282, Approval date: April 24, 2022), and informed consent was obtained from all participants before their participation.

### Route Navigation Task

**Figure 1.**
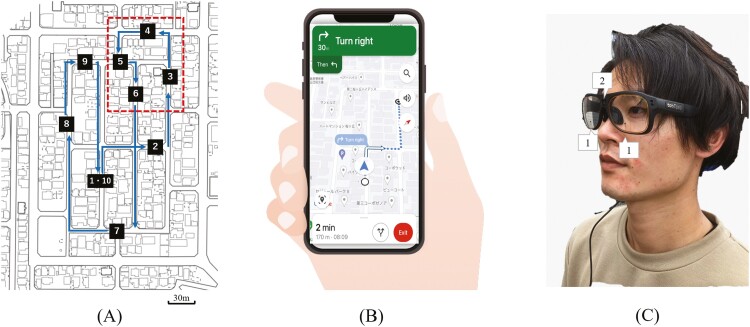
Study settings. (A) The route navigation task course used in this study: The number of times the participants glanced at their smartphones was counted from the video recordings of gaze behavior while walking the route from #3 to #6 surrounded by dotted line. (B) Participants walked with one hand holding the smartphone, following on-screen navigation. (C) Wearing an example of the Tobii Pro Glasses 3 eye tracking system, which has two cameras for eye tracking (1) and a high-definition scene camera in the center to record the forward viewpoint while walking (2).

We used the route navigation task (RNT) used in our previous study (Shimokihara et al., 2021) to assess the participants’ navigation ability using the smartphone app in an unfamiliar outdoor setting. An outdoor course of 1,300 m was designed, which the older individuals considered as a “neighbourhood outing” (Suminski et al., 2015; Figure 1A). Next, the examiner instructed the participants to walk as quickly as they could from the starting point to the destination using the navigation app without asking for directions from third parties (examiners or residents). Only when the exam administrator determined that a participant had a route error and was not aware of it in person (e.g., the participant continued to travel more than 5 m on the wrong route), the exam administrator stopped the participant and requested that he or she observe the screen carefully again, but did not teach the correct route. The examiner followed behind and supervised the participants to ensure their safety and rapid action in case of emergency or unforeseen circumstances. The mobile devices used in this study were those routinely used by the participants and were updated to the latest versions of the operating system (iOS or Android) and navigation app (Google Maps). When participants needed to install or update a new navigation app for RNT, they used an internet connection in the laboratory. The navigation app was configured by the examiner to always display upward directions during the navigation walk (Figure 1B). The cost of internet communication during the RNT was borne by the participants (approximately 20 MB of data for 20 min of use of the navigation application). The outcomes of RNT were total walking time (s), number of stops (times), and number of route errors (times), as recorded by the examiner accompanying the participant. Because the total walking time of RNT includes both stop time and route error time, it was used as a parameter to represent the efficiency of the app-based navigation walk.

### Evaluation of Gaze Behavior

Tobii Pro Glasses 3 and Tobii Pro Lab software (Tobii Technology, Danderyd, Sweden) were used to assess the participants’ gaze behavior during navigation walking (Figure 1C). Tobii Pro Glasses 3, a system that records binocular gaze direction, also provides pupil size, the 3D orientation of each eyeball in a coordinate system fixed to the headset, and gyroscope and accelerometer data indicating the movement of the headset. The sampling frequency of the eye tracker was 100 Hz. The Tobii Pro Glasses series is a head-worn eye tracker used in various studies (Koletsis et al., 2017; Rogers et al., 2018; Topolšek et al., 2016). Fixations and saccades were detected using the Tobii Pro Lab default I-VT filter (velocity threshold: 30°, minimum fixation duration: 60 ms). In this study, we used the following gaze information data extracted from the raw data collected by the eye tracker using dedicated analysis software: total duration of whole fixations (s), average duration of whole fixations (ms), number of whole fixations (times), number of saccades (times), average peak velocity of saccades (degrees/s), average amplitude of saccades (degrees), total amplitude of saccades (degrees). Additionally, from the simultaneously recorded gaze behavior movie data from the eye tracker, we quantified the number of times participants glanced at their smartphones while walking the course from #3 to #6 counted after the RNT. Supplementary Figure 1 shows the evaluation procedure for the number of glances.

### Assessment of Mobile Device Use and Proficiency

The Short Version of the Mobile Device Proficiency Questionnaire (MDPQ-16) assessed proficiency in eight areas related to mobile devices: the fundamentals of using a mobile device, communication, data and file storage, the internet, calendar, entertainment, privacy and troubleshooting, and software management. The MDPQ-16 uses a 5-point Likert scale and the questionnaires were scored as previously described, and had a high internal consistency (Moret-Tatay et al., 2019; Roque & Boot, 2018).

Moreover, this study also assessed which apps participants were using. These survey items were based on the Telecommunication Usage Trend Survey conducted by the Ministry of Internal Affairs and Communications in Japan (*Results of FY2019 Communication Usage Trend Survey**|**Press Release*, n.d.). The groupings were as follows: (a) messages/texting, (b) email, (c) phone calls, (d) internet browsers, (e) cameras, (f) social networking services, (g) video viewing, (h) online games, (i) news and weather, (j) maps/navigation, (k) online shopping, (l) financial transactions, (m) contents, (n) auctions, (o) electronic government communication. Participants were also asked to indicate the number of years they had been using their mobile devices.

### Exploration of Factors Associated With Navigation Ability in Older Adults

#### Japan Science and Technology Agency Index of Competence

We used the Japan Science and Technology Agency Index of Competence (JST-IC) to assess functional capacity. The JST-IC covers four domains (technology usage, information practice, life management, and social engagement) and assesses higher levels of daily functioning adapted to modern life. The higher the score, the greater the individual’s competence in daily life. Each domain consists of four items that are scored on a dichotomous rating scale (0 or 1), yielding subscores of 0–4 and a total score of 0–16, with higher scores indicating greater competence in daily life. The Cronbach’s alpha coefficient for all 16 items is 0.86 (Iwasa et al., 2018), which is reliable and has been used in many studies on the older Japanese population (Akasaki et al., 2022; Maruta et al., 2020; Shoji et al., 2022; Wada et al., 2022).

#### Life-space assessment

The Japanese version of the Life-Space Assessment (LSA; Harada et al., 2010) was used to measure life-space mobility. The LSA is a self-report measure that asks participants to quantify how far and how often they have moved, with or without assistance, in the past 4 weeks. The LSA is used to assess an individual’s mobility pattern across five levels of their living space (bedroom, home, outside house, neighborhood, and town) during the last 4 weeks. Participants were asked how frequently they moved or traveled within a specific area for each of the five life-space levels and whether they needed assistance from another person or an assistive device. The LSA scores are calculated for a range from living space limited to the bedroom (LSA = 0) to daily outings without equipment or assistance (LSA = 120; Peel et al., 2005). The LSA has been shown to be a valid and reliable measure among participants recruited from a variety of cultures (Al Snih et al., 2012; Curcio et al., 2013; Fristedt et al., 2016; Ji et al., 2015; McCrone et al., 2019; Portegijs et al., 2014).

#### Cognitive function

The Japanese version of the Mini-Mental State Examination (MMSE-J) was used to measure participants’ cognitive function. The MMSE is a commonly used set of questions for screening cognitive function (Folstein et al., 1975). The MMSE-J is a brief, quantitative measure of cognitive status in Japanese older adults. The MMSE-J is reported to be equivalent to the original version of the MMSE (M. Sugishita et al., 2010). The MMSE-J can be used to assess six areas of cognitive status, including orientation to time and place, attention or concentration, short-term memory (recall), language skills, visuospatial abilities, and ability to understand/follow instructions. The MMSE-J consists of 11 questions or tasks, and the score is the number of correct items. The maximum score is 30. A score of 26 or higher is considered normal (Chun et al., 2021), while a score of 23 or less is generally accepted as a cutoff point indicating the presence of cognitive impairment or dementia (H. Li et al., 2016). The examination has been validated in a number of populations.

#### Geriatric Depression Scale

The 15-item version of the Geriatric Depression Scale (GDS-15) was used to measure mood symptoms (Brink et al., 1982). The assessment is suitable for screening mood symptoms in community-dwelling older adults (De Craen et al., 2003). Each item requires a dichotomous response (*yes* or *no*) that is scored as 1 or 0, respectively. Scores range from 0 to 15, with higher scores indicating greater severity of mood symptoms. The reliability and validity of the GDS-15 have been verified in older Japanese people (K. Sugishita et al., 2017).

#### Walking speed test

Maximum gait speed was measured on a 15 m walkway in the laboratory. The initial and final 2.5 m sections were not recorded to allow for acceleration and deceleration.

#### Demographic factors

Data were collected on sociodemographic variables, including age (years), sex, and education (years) as potential factors that correlate with app-based navigation walk.

### Procedure

All participants participated in one session, which comprised completing the consent form, paper assessment and physical assessment, gaze sensor fitting, and RNT. The session lasted approximately 120 or 90 min for the older and the younger participants, respectively. At the start of the RNT, participants were shown the purpose of the task and a tutorial. After completing a tutorial, participants began an app-assisted navigation walk; the session ended when the RNT was completed. In addition, internet connection costs during the RNT were covered by the participants (approximately 20 MB of data for 20 min of navigation app use). Whenever it was necessary to install or update a new navigation application for the RNT, we used an internet connection in the laboratory.

### Statistical Analysis

For comparisons between the older participants and the young participants, appropriate statistical tests were used depending on the normality of the continuous variables. Specifically, the Shapiro–Wilk test was used to test the normality assumption of the variables. A parametric test (unpaired *t* test) or nonparametric test (Mann–Whitney U test) was then used to compare variables. Categorical variables were compared between the older and younger groups by using Pearson’s Chi-squared test or Fisher’s exact test. Approximate effect sizes (ES) were provided for inferential tests. In addition, a series of univariate regression analyses were conducted to determine the association between the RNT outcomes and age. Subsequently, to examine factors associated with app-based navigation walk performances in older participants, the general linear regression models were used to assess the statistical significance of the associations between the performances of app-based navigation walk and the clinical variables. The regression models were created with RNT performance as the dependent variables, specifically efficiency and accuracy (number of stops and route errors) and the parameters of gaze behavior that were significantly different between older and young participants. Demographic variables, paper assessment scores, and gait speed were set as the independent variables. R version 4.2.2 was used for all statistical analysis and plotting. For all tests, statistical significance was set at *p* < .05.

## Results

### Differences in App-Based Navigation Ability Between Older and Young Participants

**Figure 2.**
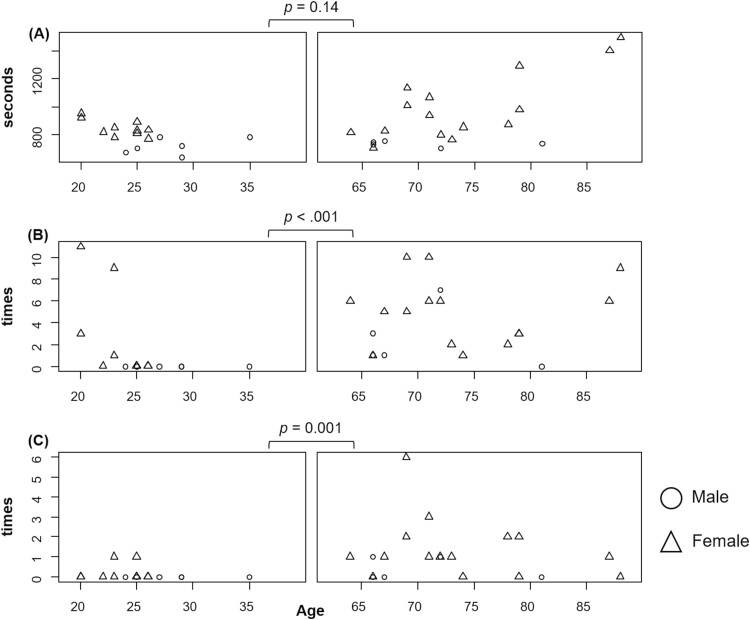
Comparison of route navigation task outcomes. (A) total duration of route navigation task, (B) number of stops, and (C) number of route errors. All statistics were performed by Mann–Whitney U test.

Twenty community-dwelling older adults and 16 healthy young adults participated in the study without any unforeseen events. The mean age of the older participants was 73.5 ± 8.1 years (female participants 75.0%), and the mean age of the younger participants was 25.3 ± 3.7 years (female participants 62.5%). Figure 2 compares the RNT outcomes between older and young participants. The older participants had significantly more stops (Figure 2B, ES = 0.57) and route errors (Figure 2C, ES = 0.54) in the RNT than the young participants. Moreover, older participants reported significantly fewer years of mobile device use than younger participants (older adults: 6.0 ± 5.5; young adults: 9.4 ± 1.7 [years], *t* = 2.41, *p* = .02, ES = 0.79).

### Characteristics of Gaze Behavior During App-Based Navigation Walking

**Figure 3.**
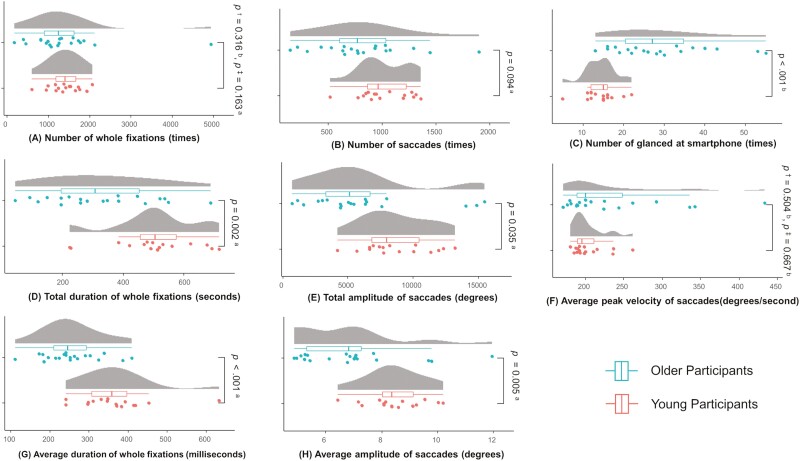
Comparison of gaze behavior during route navigation task. Comparisons of gaze behavior outcomes during app-based navigation walking: statistical test performed; ^a^unpaired *t* test, ^b^Mann–Whitney U test. *p*^†^ denotes the value of the comparison result including outliers and *p*^‡^ denotes the value of the comparison result excluding outliers.

A comparison of outcomes related to gaze behavior is shown in Figure 3. Older participants had significantly more glances at their smartphones while walking a particular route than young participants did (Figure 3C, ES = 0.72). Furthermore, the total duration of whole fixations (Figure 3D, ES = 1.08), total amplitude of saccades (Figure 3E, ES = 0.72), average duration of whole fixations (Figure 3G, ES = 1.32), and average amplitude of saccades (Figure 3H, ES = 0.99) were significantly less in the older participants than in the young participants. Two variables with extreme outliers were detected in the distribution of the data (number of whole fixations, and average peak velocity of saccades). The results remained after excluding the outliers and reanalyzing (Figure 3A, *p* = .163; Figure 3F, *p* = .667). The results showed that older adults had to look at their smartphone more often to check it while walking with the navigation app compared with young participants, but did not receive enough information from the app or the environment due to the shorter fixation time. In addition, the decrease in saccade amplitude of the older participants suggests that they had a narrower field of view during navigation walking.

### Age-Related Effects of Navigation Ability and Gaze Behavior in App-Based Navigation Walking

Supplementary Table 1 shows the results of a series of univariate regression analyses for age and RNT outcomes. Age was significantly positively associated with increased duration (β = 0.38) and number of route errors (β = 0.44) in the app-based navigation walking. The number of glances at the smartphone during the app-based navigation walk increased significantly with age, whereas the duration of fixation and the number and angle of saccades decreased significantly with age. The number of glances at the smartphone, average fixation duration, and total fixation duration were highly associated with age (β = 0.59, −0.55, and − 0.50, respectively).

### Mobile Device Proficiency and Application Usage by Older and Young Adults

Older participants were found to have significantly lower MDPQ-16 scores than young participants (*p* = .003, ES = 0.58). Table 1 shows a comparison of the percentage of apps used on smartphones between the older and younger groups. The use of the following apps was significantly less among the older group than the young group: social networking services, video viewing, online games, maps or navigation, online shopping, financial transactions, and contents.

**Table 1.** Types of Smartphone Applications Used by Study Participants

Smartphone application type	Older participants *n* = 20	Young participants *n* = 16	Effect size	*p* Value
	*n* (%)	*n* (%)		
1. Messages/texting (e.g., Facebook Messenger, WhatsApp, and Line)	18 (90.0)	16 (100.0)	0.22	0.49^b^
2. Email (e.g., Gmail and Outlook)	19 (95.0)	14 (87.5)	0.13	0.57^b^
3. Phone calls	17 (85.0)	13 (81.3)	0.05	0.99^b^
4. Internet browsers (e.g., Safari and Chrome)	12 (60.0)	9 (56.3)	0.04	0.82^a^
5. Cameras	18 (90.0)	16 (100.0)	0.22	0.49^b^
6. Social networking services (e.g., Facebook, Twitter, and Instagram)	10 (50.0)	15 (93.8)	0.47	0.009^b^
7. Video viewing (e.g., YouTube and Netflix)	11 (55.0)	16 (100.0)	0.52	0.002^b^
8. Online games	3 (15.0)	10 (62.5)	0.49	0.003^a^
9. News and Weather	18 (90.0)	16 (100.0)	0.22	0.49^b^
10. Maps/navigation	13 (65.0)	16 (100.0)	0.44	0.01^b^
11. Online shopping (e.g., Amazon and Rakuten)	9 (45.0)	15 (93.8)	0.51	0.002^a^
12. Financial transactions	3 (15.0)	12 (75.0)	0.61	<.001^a^
13. Contents (e-books and music)	3 (15.0)	12 (75.0)	0.61	<.001^a^
14. Auctions	3 (15.0)	7 (43.8)	0.32	0.07^b^
15. Electronic government communication (e.g., tax payment and address change)	6 (30.0)	4 (25.0)	0.06	0.99^b^

*Notes*: Statistical test performed:

^a^Chi-square test of independence.

^b^Fisher’s exact test.

### Factors Associated With Older Participants’ App-Assisted Navigation Walk

The results of the multivariable general linear regression analyses are shown in Table 2. Among the older participants, the total walking time of the app-based navigation walk was associated with walking speed (*p* = .03), LSA (*p* < .001), MMSE-J (*p* = .01), and MDPQ-16 (*p* = .02). No variables were found to be significantly associated with the number of stops or route errors during RNT. In Supplementary Table 2, we investigated associations by performing multiple regression models for the five items (number of glances at smartphone, total duration of whole fixations, total amplitude of saccades, average duration of whole fixations, and average amplitude of saccades) that showed significant differences from young participants in gaze behavior during the RNT. The results showed that no factors were significantly associated with the gaze behavior of the older participants.

**Table 2.** Factors Associated with App-Based Navigation Gait in Older Participants

Factors	Mean ± *SD*	Models	Estimate	β	95% CI		*t* Value	*p* Value
					Lower	Upper		
Age (years)	73.5 ± 8.1	Model 1	−2.419	−0.09	−0.62	0.44	0.38	.13
		Model 2	−0.07	−0.14	−1.86	1.57	−0.19	.86
		Model 3	−0.19	−0.89	−2.62	0.85	−1.14	.28
Education (years)	12.9 ± 1.8	Model 1	36.528	−0.03	−0.48	0.43	0.15	.88
		Model 2	0.22	0.13	−0.73	0.99	0.33	.75
		Model 3	−0.05	−0.06	−0.93	0.81	−0.16	.88
Sex, *n* (%) (ref.: female)	15 (75.0)	Model 1	−74.689	−0.50	−1.26	0.26	1.46	.18
		Model 2	−1.48	−0.47	−2.84	1.89	−0.45	.66
		Model 3	−0.55	−0.38	−2.77	2.00	−0.36	.73
Gait speed (m/s)	1.8 ± 0.4	Model 1	−145.814	−0.76	−1.43	−0.11	2.60	.03
		Model 2	−0.32	−0.04	−1.21	1.14	−0.08	.94
		Model 3	−0.94	−0.26	−1.45	0.93	−0.49	.64
JST−IC (score)	12.5 ± 3.8	Model 1	20.468	0.02	−0.54	0.57	0.07	.95
		Model 2	0.33	0.31	−1.27	1.90	0.44	.67
		Model 3	0.05	0.11	−1.49	1.71	0.15	.88
LSA (score)	111.5 ± 36.1	Model 1	−7.957	−1.23	−1.93	−0.53	3.91	<.001
		Model 2	−0.02	−0.22	−1.38	0.95	−0.41	.69
		Model 3	0.00	0.04	−1.14	1.22	0.07	.94
GDS (score)	1.4 ± 1.5	Model 1	−0.582	−0.03	−0.29	0.22	0.30	.77
		Model 2	0.67	0.32	−0.27	0.92	1.21	.26
		Model 3	0.18	0.19	−0.41	0.79	0.70	.50
MMSE-J (score)	27.7 ± 2.1	Model 1	−18.013	−0.68	−1.15	−0.20	3.18	.01
		Model 2	−0.46	−0.32	−1.05	0.42	−0.95	.36
		Model 3	−0.23	−0.35	−1.09	0.40	−1.04	.32
MDPQ-16 (score)	31.7 ± 8.9	Model 1	−2.443	−0.58	−1.04	−0.11	2.64	.02
		Model 2	−0.02	−0.13	−1.65	1.39	−0.19	.85
		Model 3	−0.03	−0.50	−2.04	1.03	−0.73	.48

*Notes*: β = standardized partial regression coefficient; CI = confidence interval; GDS = 15-item version of the Geriatric Depression Scale; JST-IC = Japan Science and Technology Agency Index of Competence; LSA = Life-Space Assessment; MDPQ-16 = Short Version of the Mobile Device Proficiency Questionnaire; MMSE-J = Japanese version of the Mini-Mental State Examination; RNT = route navigation task; *SD* = standard deviation.

General linear model: Model 1, dependent variable; total duration of RNT, adjusted *R*^2^ = 0.88, overall model test; *F* = 15.13, *p* < .001, power = 0.95. Model 2, dependent variable; number of stops in RNT, adjusted *R*^2^ = −0.26, overall model test; *F* = 0.57, *p* = .79. Model 3, dependent variable; number of route errors in RNT, adjusted *R*^2^ = −0.28, overall model test; *F* = 0.54, *p* = .82.

## Discussion

The study aimed to compare accuracy and gaze behavior between older and young participants in a real-world app-based navigation walk and examine the clinical factors affecting app-based navigation in older adults. The results showed efficiency and gaze behavior during app-based navigation walking which is characteristic of older adults. There was a significant relationship between the efficiency of app-based navigation walking and walking speed, cognitive function, life space, and proficiency with mobile devices in older adults. These findings highlight the adaptations and limitations for older adults in using navigation apps in the real world to achieve independent mobility and are promising for future navigation assistance research.

### Age Differences in Gaze Behavior During App-Based Navigation Walk

Gaze behavior characteristics during the app-based navigation walk revealed that older participants had lower total and mean fixation times than young participants. Moreover, older participants had a lower mean amplitude and total angle of saccade than young participants. It was also noted that older participants spent less time fixating and tended to glance at their smartphones more often during navigation walking. This result was maintained in a univariate regression model with age as the independent variable. Though there are few studies on gaze behavior during navigation walks, research conducted by Irving et al. (2018) found an increase in the total number of saccades among older adults, which was linked to a shift toward egocentric strategies. Additionally, the decrease in saccade amplitude among older participants is reflective of a decrease in their effective field of vision (Shih et al., 2012; Veiel et al., 2006), indicating that young participants have better visual memory (D’Innocenzo et al., 2022). The RNT may require frequent switching from the allocentric strategy of navigation app screens to the egocentric strategy of moving in the real world. It has been suggested that switching from the allocentric to the egocentric strategy is particularly difficult for older adults (Colombo et al., 2017). Our results suggest that older adults may have had to work harder to use the two strategies during app-based navigation walking, and may have had to focus on the smartphone screen and a limited range of gaze movements in the direction of travel. The lower fixation time may also be due to age-related decline in their ability to process information as certain cognitive domains deteriorate with age (Salthouse, 2004; Schaie, 2013). Young participants acquired more information per fixation and may have been better at guessing the route than older participants. Therefore, the older participants must have had to repeatedly glance at their mobile devices during navigation walking to confirm the route. The results of the study suggest that older participants may have had difficulty recognizing targets such as buildings and signs during navigation, as indicated by a higher number of stops and route errors. Therefore, when providing navigation assistance to older adults, a more detailed description of the target object when changing direction should be required.

### Adaptation of Navigation Apps for Older Adults

Older adults spent 46.3% of their smartphone app activations on communication apps and 2.6% on navigation or map apps (Gordon et al., 2019). In this study, our results also showed significantly less navigation app use for older participants than for young participants, inferring that navigation app use for older adults is not common practice. The app-based navigation system consisted of an on-screen map with illustrations of arrows, distances and building names in text form, and audio guidance. Older adults prefer egocentric navigation strategies (Bécu et al., 2020; Rosenbaum et al., 2012), but have difficulty switching navigation strategies (Harris & Wolbers, 2014). Navigation app functions may contribute to the egocentric strategy by showing the direction of travel from the current location with arrows and audio. In addition, displaying the current location on an overhead map and showing the location and names of landmarks may support the allocentric strategy. Thus, the navigation app could support both egocentric and allocentric strategies. However, age-related hearing and vision impairments are common among older adults (Bourne et al., 2017; Lin et al., 2011), and many apps are not specifically designed for their use, requiring improvements in usability issues such as text size and volume of voice prompts are needed. In fact, our results showed that older adults are less proficient with mobile devices than younger participants. Recently, research has shown that voice-only guidance is effective for navigation while driving for people with mild Alzheimer’s disease (Yi et al., 2015), and haptic-based walking guidance has been explored for people with visual or hearing impairments (Barontini et al., 2021; Sorgini et al., 2018). Particularly, navigation assistance for older adults using an egocentric strategy is more acceptable, less error-prone, and likely to be more efficient (Rodgers et al., 2012). Therefore, further research could investigate the most appropriate setting for walking guidance for older adults.

### Clinical Associated With App-Based Navigation Walk in Older Adults

This study found that walking speed, cognitive function (as measured by the MMSE-J), life-space (as measured by the LSA), and proficiency with mobile devices (as measured by the MDPQ-16) are associated with efficient app-based navigation walking in older adults. Regarding the MMSE-J being related to the app-based navigation walk, because the RNT comprised a dual-task, we thought a possibility was that cognitive and physical functions, such as attention and executive function, were required to complete the task, which reduced walking speed and increased the time to complete the task (Raichlen et al., 2020; Schwenk et al., 2010; Verghese & Holtzer, 2010). Future research should examine the factors associated with app-based navigation walks in combination with an assessment of higher brain function. Regarding LSA, De Silva et al. (2019) showed that cognitive decline in older adults with limited living space, and Dunlap et al. (2021) found a significant relationship between LSA scores and gait-related measures. Considering the relationship between walking speed, cognitive function and LSA, efficient app-based navigation and life space may also have a bidirectional relationship. In other words, older adults who use navigation apps more efficiently may live in a wider range of situations. Moreover, the study also showed a wide variability in the use of mobile devices among older adults. Although smartphone use among older adults is growing rapidly and digital technology is becoming more widely accepted (Mitzner et al., 2010), it is important to understand that there is a wide range of proficiency among the older population (Moret-Tatay et al., 2019). Note that the MDPQ-16 does not include items that directly assess the use of navigation apps, but it does include basic items related to the use of mobile devices, so the general ability of older participants to use mobile devices may also be related to app-based navigation walks. Summarizing earlier, the prevention of physical and cognitive decline, the retention of a wide range of living spaces, and the improvement of mobile device proficiency might be addressed early in the aging process. These efforts could help older adults adapt digital technology to their daily lives and remain independent and outgoing in the community.

### Limitations and Future Perspectives

The results of this study should be understood while considering several limitations. First, the community-dwelling older adults in this study were recruited from the SHRC; older adults who participate in the SHRC are likely to be more physically and cognitively robust than the general older population. Therefore, when adapting the results of this study to the general older population, considering the possibility of them having lower physical and cognitive function than the older adults in this study is necessary. By contrast, if the results of this study are viewed as data from older adults before the decline in cognitive and physical functions progresses, the results may clarify the functions required for app-based mobility support using mobile devices to prevent functional decline among the older population. Second, an evaluation of the financial status was missing. Previous reports have shown that smartphone ownership varies by income and is particularly high among higher-income groups (Laricchia, 2022). Therefore, it is possible that smartphone owners are in a better financial status than those who do not own a smartphone. Future research should include additional assessments of financial status. Third, the number of participants in this study was relatively small. Therefore, the effect of age may increase in a larger sample than in this study. In addition, older participants should be more adept at egocentric strategies than young participants because they have a better understanding of the environment around their neighborhoods where they have lived for many years. The current method may have underestimated the potential of older adults because the RNT was only tested using a route that was unfamiliar to the participants. Further work on the performance of app-based navigation, including familiar routes, would help to support older adults in using their mobile devices to freely go out. Last, the number of glances at the smartphone was assessed by a single examiner. The obtained mean and standard error were 22.5 ± 1.96. Although the margin of error is small, the possibility of systematic errors depending on the evaluator cannot be ruled out. Even with these limitations, this study proposes a navigation support strategy that is more appropriate for older adults based on age-related differences in gaze behavior of app-based navigation support that have not been previously clarified. Furthermore, it is noteworthy that the factors associated with app-based navigation walk suggest that older adults may be able to adapt digital technology to freely navigate outdoors in the future.

## Conclusion and Implications

This study revealed differences in accuracy and gaze behavior with age when walking with an app that assists navigation in the real-world. Additionally, the relationship between clinical variables and efficient app-based navigation walking among older adults was investigated. The results suggest that older adults make more stops and route errors than young adults during app-based navigation walking and may have difficulty switching perspectives between the smartphone screen and the real world. They also had lower fixation time and saccade amplitude and angle, suggesting their effective visual field during app-based walking may be smaller. In addition to basic functions such as walking speed and cognitive function, the range of life and proficiency with mobile devices were associated with the efficiency of app-based navigation walking in older adults. These findings provide a basis for further research, including the development of a new navigation system to support the independent mobility of older adults, and the need to understand the level of proficiency of older adults with mobile devices.

## Supplementary Material

igad108_suppl_Supplementary_Figures_S1_Tables_1-2
