# Supplementary material for: Real-World Navigation With Application: Characteristics of Gaze Behavior and Associated Factors in Older Adults
Source: Innov Aging. 2023 Sep 21;7(8):igad108. doi: 10.1093/geroni/igad108 (PMC10630010; doi:10.1093/geroni/igad108)
Supplement: igad108_suppl_Supplementary_Figures_S1_Tables_1-2 [file igad108_suppl_supplementary_figures_s1_tables_1-2.docx]

Online Supplementary Material

Table S1

*Univariate regression analyses of RNT outcomes and age*

| RNT outcome | adjusted R^2^ | Estimate | t | β | 95% CI  (lower, upper) | *p*-value |
| --- | --- | --- | --- | --- | --- | --- |
| Total duration of RNT (sec) | 0.12 | 2.61 | 2.37 | 0.38 | 0.05, 0.71 | 0.02 |
| Number of stops (times) | 0.11 | 0.05 | 2.01 | 0.33 | -0.01, 0.67 | 0.33 |
| Number of route errors (times) | 0.17 | 0.02 | 2.78 | 0.44 | 0.12, 0.75 | 0.01 |
| Number of glanced at smartphone (times) | 0.33 | 0.29 | 4.23 | 0.59 | 0.31, 0.88 | <.001 |
| Number of whole fixations (times) | 0.02 | -4.25 | -1.29 | -0.22 | -0.56, 0.13 | 0.21 |
| Average duration of whole fixations (ms) | 0.28 | -2.15 | -3.79 | -0.55 | -0.85, -0.26 | <.001 |
| Total duration of whole fixations (sec) | 0.23 | -3.77 | -3.31 | -0.50 | -0.81, -0.19 | 0.002 |
| Number of saccades (times) | 0.13 | -5.27 | -2.45 | -0.39 | -0.72, -0.07 | 0.02 |
| Total amplitude of saccades (degrees) | 0.16 | -64.5 | -2.74 | -0.43 | -0.75, -0.11 | 0.01 |
| Average amplitude of saccades (degrees) | 0.19 | -0.03 | -3.03 | -0.47 | -0.78, -0.15 | 0.005 |
| Average peak velocity of saccades (degrees/sec) | 0.02 | 0.46 | 1.29 | 0.22 | -0.13, 0.56 | 0.21 |
| Notes. Β=standardized partial regression coefficient; CI=confidence interval, RNT=route navigation task. Regression models were constructed with each outcome as the dependent variable and age as the independent variable. | | | | | | |

Table S2

*Factors associated with gaze behavior during app-based navigation gait in older participants*

| Factors | Models | Estimate | β | 95% CI | | t | *p*-value |
| --- | --- | --- | --- | --- | --- | --- | --- |
|  |  |  |  | Lower | Upper |  |  |
| Age (years) | Model 1 | 0.04 | 0.03 | -1.67 | 1.72 | 0.03 | 0.97 |
|  | Model 2 | 23103.00 | 0.90 | -0.43 | 2.23 | 1.51 | 0.16 |
|  | Model 3 | -11.66 | -1.08 | -2.65 | 0.49 | -1.53 | 0.16 |
|  | Model 4 | 641.80 | 1.02 | -0.33 | 2.36 | 1.69 | 0.12 |
|  | Model 5 | 0.14 | 0.52 | -1.11 | 2.14 | 0.71 | 0.50 |
| Education (years) | Model 1 | 1.60 | 0.25 | -0.59 | 1.10 | 0.66 | 0.52 |
|  | Model 2 | 32776.00 | 0.35 | -0.31 | 1.02 | 1.18 | 0.27 |
|  | Model 3 | -9.97 | -0.25 | -1.04 | 0.53 | -0.72 | 0.49 |
|  | Model 4 | 1023.10 | 0.45 | -0.22 | 1.12 | 1.49 | 0.17 |
|  | Model 5 | 0.19 | 0.19 | -0.62 | 1.00 | 0.53 | 0.61 |
| Sex, n (%)  (ref: female) | Model 1 | -1.17 | -0.10 | -2.43 | 2.24 | -0.09 | 0.93 |
|  | Model 2 | -91837.00 | -0.53 | -2.36 | 1.30 | -0.65 | 0.53 |
|  | Model 3 | -17.67 | -0.24 | -2.41 | 1.92 | -0.25 | 0.81 |
|  | Model 4 | -446.30 | -0.10 | -1.96 | 1.75 | -0.13 | 0.90 |
|  | Model 5 | -0.13 | -0.07 | -2.31 | 2.16 | -0.07 | 0.94 |
| Gait speed (m/sec) | Model 1 | 12.21 | 0.40 | -0.76 | 1.56 | 0.78 | 0.46 |
|  | Model 2 | 22912.00 | 0.05 | -0.86 | 0.96 | 0.13 | 0.90 |
|  | Model 3 | -79.37 | -0.43 | -1.50 | 0.65 | -0.88 | 0.40 |
|  | Model 4 | 683.10 | 0.06 | -0.86 | 0.98 | 0.15 | 0.88 |
|  | Model 5 | -0.18 | -0.04 | -1.15 | 1.07 | -0.08 | 0.94 |
| JST-IC (score) | Model 1 | 1.21 | 0.30 | -1.26 | 1.87 | 0.43 | 0.68 |
|  | Model 2 | 16946.00 | 0.29 | -0.94 | 1.52 | 0.52 | 0.61 |
|  | Model 3 | 0.39 | 0.02 | -1.44 | 1.47 | 0.02 | 0.98 |
|  | Model 4 | 758.30 | 0.52 | -0.72 | 1.77 | 0.94 | 0.37 |
|  | Model 5 | 0.14 | 0.22 | -1.28 | 1.72 | 0.33 | 0.75 |
| LSA (score) | Model 1 | 0.01 | 0.02 | -1.14 | 1.17 | 0.03 | 0.97 |
|  | Model 2 | 156.00 | 0.03 | -0.88 | 0.93 | 0.07 | 0.95 |
|  | Model 3 | 1.39 | 0.57 | -0.50 | 1.64 | 1.18 | 0.26 |
|  | Model 4 | -40.20 | -0.28 | -1.20 | 0.64 | -0.68 | 0.51 |
|  | Model 5 | 0.02 | 0.24 | -0.86 | 1.35 | 0.49 | 0.63 |
| GDS (score) | Model 1 | 2.11 | 0.27 | -0.32 | 0.86 | 1.01 | 0.34 |
|  | Model 2 | 51256.00 | 0.44 | -0.02 | 0.90 | 2.14 | 0.06 |
|  | Model 3 | 8.47 | 0.17 | -0.37 | 0.72 | 0.71 | 0.49 |
|  | Model 4 | 903.20 | 0.32 | -0.15 | 0.78 | 1.52 | 0.16 |
|  | Model 5 | 0.41 | 0.34 | -0.23 | 0.90 | 1.33 | 0.21 |
| MMSE-J (score) | Model 1 | -1.50 | -0.27 | -1.00 | 0.46 | -0.83 | 0.43 |
|  | Model 2 | -29624.00 | -0.37 | -0.94 | 0.20 | -1.43 | 0.18 |
|  | Model 3 | -8.32 | -0.24 | -0.92 | 0.43 | -0.81 | 0.44 |
|  | Model 4 | -787.30 | -0.40 | -0.97 | 0.18 | -1.53 | 0.16 |
|  | Model 5 | -0.38 | -0.44 | -1.13 | 0.26 | -1.40 | 0.19 |
| MDPQ-16 (score) | Model 1 | -0.06 | -0.11 | -1.61 | 1.39 | -0.16 | 0.88 |
|  | Model 2 | 4913.00 | 0.64 | -0.54 | 1.81 | 1.20 | 0.26 |
|  | Model 3 | -2.07 | -0.64 | -2.03 | 0.76 | -1.02 | 0.33 |
|  | Model 4 | 131.30 | 0.69 | -0.50 | 1.88 | 1.29 | 0.23 |
|  | model 5 | 0.04 | 0.47 | -0.96 | 1.91 | 0.74 | 0.48 |
| Notes. Β=standardized partial regression coefficient; CI=confidence interval, GDS=15-item version of the Geriatric Depression Scale, JST-IC=Japan Science and Technology Agency Index of Competence, LSA; Life-Space Assessment, MDPQ-16=Short Version of the Mobile Device Proficiency Questionnaire, MMSE-J=Japanese version of the Mini-Mental State Examination. General linear model: Model S1, dependent variable;number of glanced at smartphone, adjusted R^2^ = −0.22, overall model test; F = 0.61, *p* = 0.76, Model S2, dependent variable; total duration of whole fixations, adjusted R^2^ = 0.25, overall model test; F = 1.69, *p* = 0.21, Model S3, dependent variable; total amplitude of saccades, adjusted R^2^ = 0.23, overall model test; F = 1.63, *p* = 0.23, Model S4, dependent variable; average duration of whole fixations, adjusted R^2^ = −0.05, overall model test; F = 0.90, *p* = 0.56, Model S5 dependent variable; average amplitude of saccades, adjusted R^2^ = −0.12, overall model test; F = 0.77, *p* = 0.65. | | | | | | | |

**Figure S1**

*Procedure of assessing for the number of times participants glanced at their smartphones*


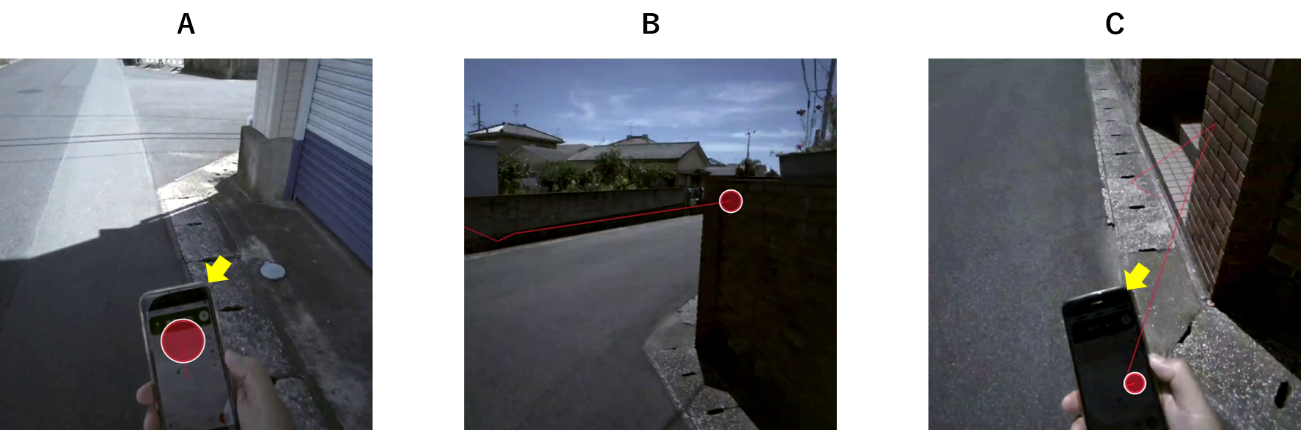


*Note.* (A) and (C) are added one additional time to the number of times they glanced at their smartphones. (B) Gaze behavior data were continuously collected even when the eyes were not looking at the smartphone screen.
